# Computer modeling and validation testing for glenoid component rotation and optimal glenoid screw angles for reverse shoulder arthroplasty in an Asian population

**DOI:** 10.1007/s00264-024-06340-z

**Published:** 2024-09-30

**Authors:** Shun Sing Martin Cheng, Colin Shing-Yat Yung, Samuel De Hoi Wong, Christopher Chun Hei Yip, Issac Jun Ren Khoo, Tsoi Wan Karen Wong, Christian Fang

**Affiliations:** 1 Department of Orthopaedics of Traumatology, Queen Mary Hospital, Pokfulam, Hong Kong; 2 Department of Orthopaedics and Traumatology, The University of Hong Kong, Pokfulam, Hong Kong; 3 Li Ka Shing Faculty of Medicine, The University of Hong Kong, Pokfulam, Hong Kong

**Keywords:** Reverse shoulder arthroplasty, Glenoid fixation, Screw length, Patient specific instrumentation, 3D modelling

## Abstract

**Purpose:**

Good initial fixation of glenoid component for reverse total shoulder arthroplasty (RTSA) relies on component placement and screw purchase in the scapula bone. This is especially difficult in an Asian population with small glenoid geometry. Optimal glenoid component roll angle and screw angulation to achieve the longest screws for best fixation has not been defined in the current literature.

**Methods:**

Computer 3D modelling of 133 scapulas with RTSA performed were analyzed to determine patient specific optimal glenoid roll angle (GRA) for the longest bi-cortical screws attainable. The cranial-caudal angle (CCA), anterior-posterior angle (APA) and lengths for the superior and inferior screws were measured. Validation testing using calculated average (CA) angles and rounded average (RA) angles to the nearest 5 degree were recomputed for each case to determine the bi-cortical screw lengths achievable. The CA and RA screw lengths were compared against patient specific modelling using paired-sample t-tests.

**Results:**

Average GRA was − 1.6°, almost perpendicular to the long axis of the glenoid and achieves an average bi-cortical screw length of 51.3 mm and 45.5 mm for the superior and inferior screws respectively. The CCA and APA were 9.1° cranial and 6.5° posterior for the superior screw and screw angulation of 11.2° caudal and 0.7° anterior for the inferior screw. Validation testing shows statistically shorter screw lengths in the CA and RA models compared to patient specific modelling (*p* < 0.01).

**Conclusion:**

Validation testing with average angles for GRA, CCA and APA demonstrates strong patient heterogeneity and anatomical variation. Despite this, screw lengths attainable in the RA group were > 38 mm with good safety profile. Surgeons may consider the additional use of navigation-assisted, or 3D printed patient specific instrumentation to optimize baseplate and screw configuration for RTSA.

**Supplementary Information:**

The online version contains supplementary material available at 10.1007/s00264-024-06340-z.

## Introduction

Reverse Total Shoulder Arthroplasty (RTSA) is indicated for irreparable massive rotator cuff tear, severe rotator cuff arthropathy, glenohumeral arthritis, and non-reconstructable proximal humerus fractures in elderly individuals [1]. However, a notable concern associated with this procedure is the potential loosening of the glenoid component, stemming from incorrect positioning and inadequate anchorage within the scapular bone [2]. In addition to ensuring the proper orientation of the glenoid base plate and preserving high-quality subchondral bone, augmenting the quantity and length of screws for the glenoid component has been proven to enhance biomechanical fixation [3–6]. It is crucial to direct each screw through the glenoid vault to the area of most bone, with the superior screw aimed towards the scapular spine confluence and inferior screw towards the scapular body to achieve maximal screw length. Stronger screw purchase hopes to achieve superior immediate implant stability and allow bony ingrowth to prolong the implant longevity [3–5]. 

There are various challenges in glenoid component placement for RTSA. Gender and ethnicity influence significant morphological and size variations of the glenoid, sometimes resulting in glenoid dimensions smaller than the available glenoid base plate, particularly evident in populations such as the Japanese, where the mean AP glenoid dimension is studied to be of an average of only 23.4 mm [7]. The naturally small and thin scapular bone in the Asian population makes the insertion of anterior and posterior screws impractical due to lack of bone. Secondly, there is a higher risk of iatrogenic fracture associated with 4 screw insertion without additional biomechanical advantage [8]. As a result, insertion of only the superior and inferior screws for the glenoid component during RTSA in small glenoid geometry is common practice. Thus, a greater accuracy for screw insertion is required. Pre-operative 3D planning with patient-specific instrumentation (PSI), computer navigation assisted surgery and robotic assisted surgery have been used to improve accuracy albeit with logistical issues, time consumption and operative costs [9–14]. 

This study seeks to establish a guide for the optimal rotation of the glenoid component and screw trajectory to facilitate the longest bi-cortical superior and inferior screw length to be used in small-sized glenoids for centers lacking the ancillary technology or with financial constraints. To date, there is no current literature describing optimal screw placement angular trajectory and glenoid roll.

## Materials and methods

A retrospective review of all CT scapula data obtained from a university hospital 3D printing facility where in-house patient-specific instrumentation (PSI) jig fabrication for RTSA is routinely practiced. All patients from 2020 to 2023, greater than 18 years old and planned for RTSA for any indication were included in this study. Informed consent was obtained for all patients and ethics approval was obtained (HKU/HA HKW IRB Reference number: UW 21–111). Delta Xtend Reverse Shoulder System (DePuy Synthes, Raynham, Massachusetts, USA) was used in all cases. The metaglene (baseplate-peg-screw complex) has a 27 mm diameter with an 8 mm central peg and allows for four variable angle-locking screws. Patients with severe bone loss defined as Walch classification grade B2, B3 and C type were excluded from the study [15]. Native excessive glenoid surface superior tilt > 10 degrees or retroversion > 10 degrees, revision or conversion surgeries from hemi-arthroplasty were also excluded. Patient demographic information and fine-cut CT scans with ≤ 1.00 mm cuts were obtained from all patients. 3D reconstruction for glenoid geometry measurements, glenoid component roll angle and screw angulation for superior and inferior variable angle screws within 15 degrees were used. The dimensional assessments were calculated using Autodesk-Meshmixer software, version 3.5.474 (Autodesk, San Rafael, CA, USA).

### Outcome measures

**Fig. 1:**
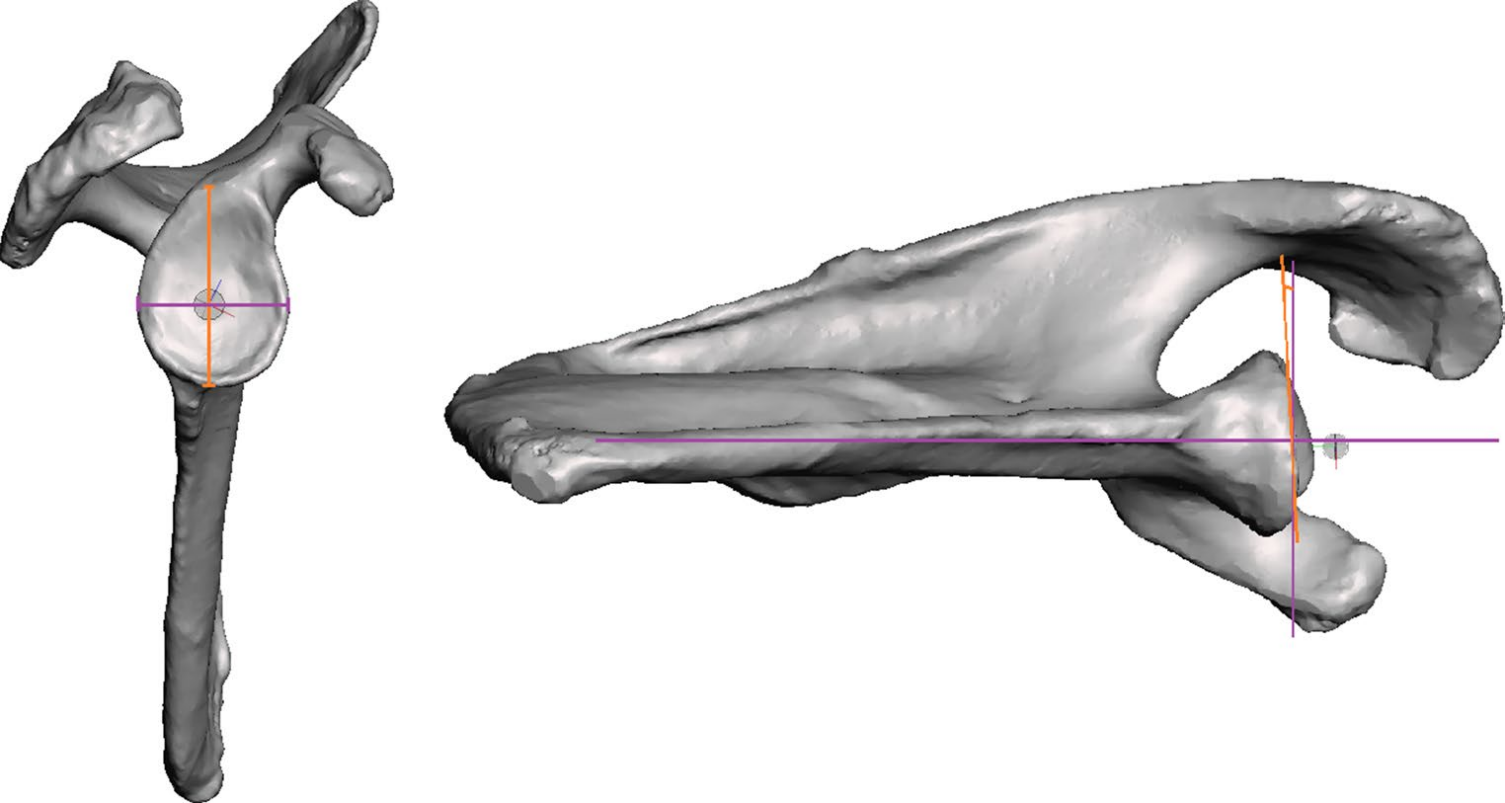
Glenoid geometrical measurements. Orange line: Cranial-caudal diameter of glenoid fossa. Purple line: Anterior-posterior width of glenoid fossa

**Fig. 2:**
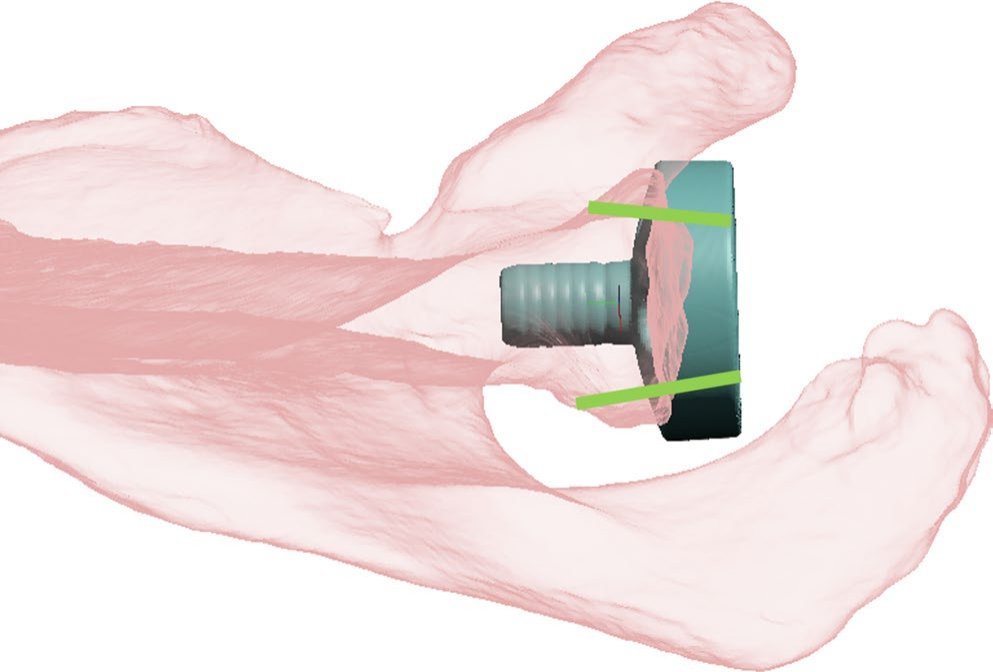
Patient specific modelling with metaglene placed nominal to the glenoid surface. Small glenoids in Asian population have inadequate or no bony purchase for anterior and/or posterior screws (green lines)

**Fig. 3:**
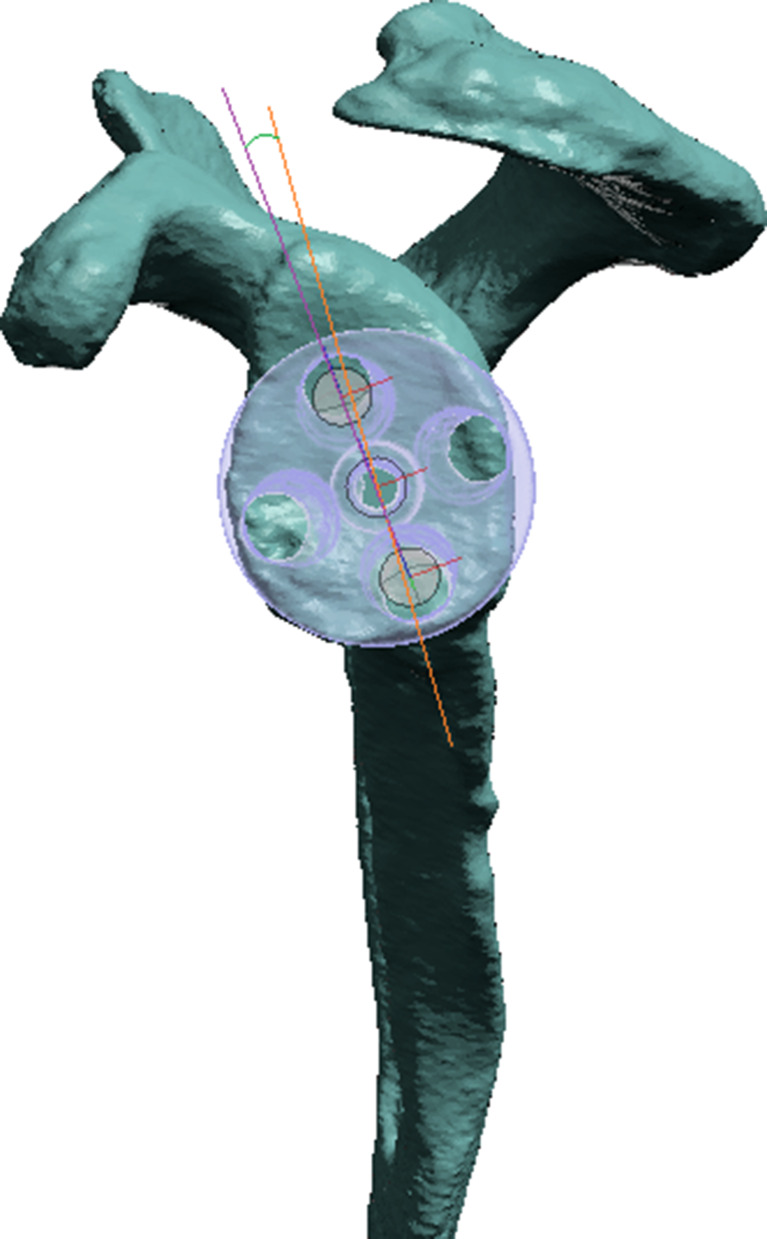
Glenoid Roll Angle (GRA) is the angle between the supraglenoid tubercle and center of metaglene line (orange) and a line intersecting the center of the superior and inferior screw holes of the metaglene representing the metaglene axis (purple)

Glenoid anterior-posterior (AP) diameter and cranial-caudal (CC) dimensions were recorded (Fig. 1). Placement of metaglenes with a tilt angle nominal to the native glenoid surface was planned to minimize reaming while also avoiding superior tilt and retroversion more than 5 degrees (Fig. 2). All metaglenes were placed at the most inferior position with its inferior margin flush to the inferior margin of the native glenoid (Fig. 3). Subsequently, rotation of the metaglene is adjusted to optimize for the longest superior screw length, ensuring its placement into the dense bone through the glenoid vault corridor congruent with the base of the scapular spine^6^. The inferior screw is adjusted to maximize bi-cortical screw length within the scapula body. The glenoid roll angle (GRA) was defined as the angle between a line drawn from the supraglenoid tubercle through the glenoid component center (glenoid axis) and a line drawn through the center of the superior and inferior screw holes (metaglene axis) as show in Fig. 3. An anterior GRA was described as positive while posterior GRA described as negative. Superior and inferior screw trajectories were referenced in relation to the central pin. Cranial-caudal angles (CCA) and anterior-posterior angles (APA) were recorded for both screws. The APA angles were described as positive and negative in the posterior and anterior direction respectively. The maximal length of bi-cortical screws were also recorded.

### Hypothesis testing and statistical analysis

To validate and investigate patient variation; the calculated averages (CA) for GRA, the superior screw angles (sCCA and sAPA), inferior screw angles (iCCA and iAPA) were re-inputted into a computer model for all cases and maximal screw lengths were recorded. These screw lengths were compared with their initial optimal screw lengths from PSI modelling using paired student t-test (SPSS version 27, Chicago, IL, USA). However, pragmatic replication of an accurate angle intra-operatively would be unrealistic. A pragmatic adaptation using rounded averages to the nearest 5 degrees for GRA, CCA and APA were also computed for each case to obtain maximal screw lengths. Statistical comparison with rounded averages (RA) screw length and initial PSI optimal screw lengths was performed using paired student t-test.

## Results

A total of 171 cases fulfilled inclusion criteria. However, 38 cases were excluded due to significant glenoid defect, revision surgery or inadequate CT scan. Analysis was performed on 133 cases. There were 99 females (74.4%) and 34 were males (24.6%). The average age was 73.6 ± 7.6 years old (range: 51–92). Most cases (62.1%) were performed for four part proximal humerus fractures. While osteoarthritis accounted for 23.4% of cases and rotator cuff arthropathy in 13.7% of cases.

In this Asian population, the glenoid geometry measurements showed an average anterior-posterior diameter of 28.2 ± 4.5 mm (range: 18.2–47.8) and a craniocaudal diameter of 38.0 ± 4.6 mm (range: 30.3–53.4). The mean glenoid version is posteriorly tilted with a mean angle of -4.13°±4.6 (range: -16.8-8.8) relative to the scapula body. Significant differences between male and female in age, AP and CC diameters as shown in Table 1 with males having larger glenoid AP and CC diameters.

**Table 1:** Patient demographics and glenoid geometrical measurements

	Female (*n* = 99)	Male (*n* = 34)	*p*-value	Combined average
Age (years ± SD)	74.6 ± 0.7	69.0 ± 1.3	**< 0.01**	73.5 ± 7.6
Glenoid A-P width (mm ± SD)	27.5 ± 3.9	31.1 ± 5.2	**< 0.01**	28.2 ± 4.5
Glenoid C-C diameter (mm ± SD)	37.1 ± 4.3	41.1 ± 4.3	**< 0.01**	38.0 ± 4.6
Glenoid Version (degree ± SD)	-4.3 ± 4.5	-3.3 ± 4.7	0.33	-4.1 ± 4.6

**Fig. 4:**
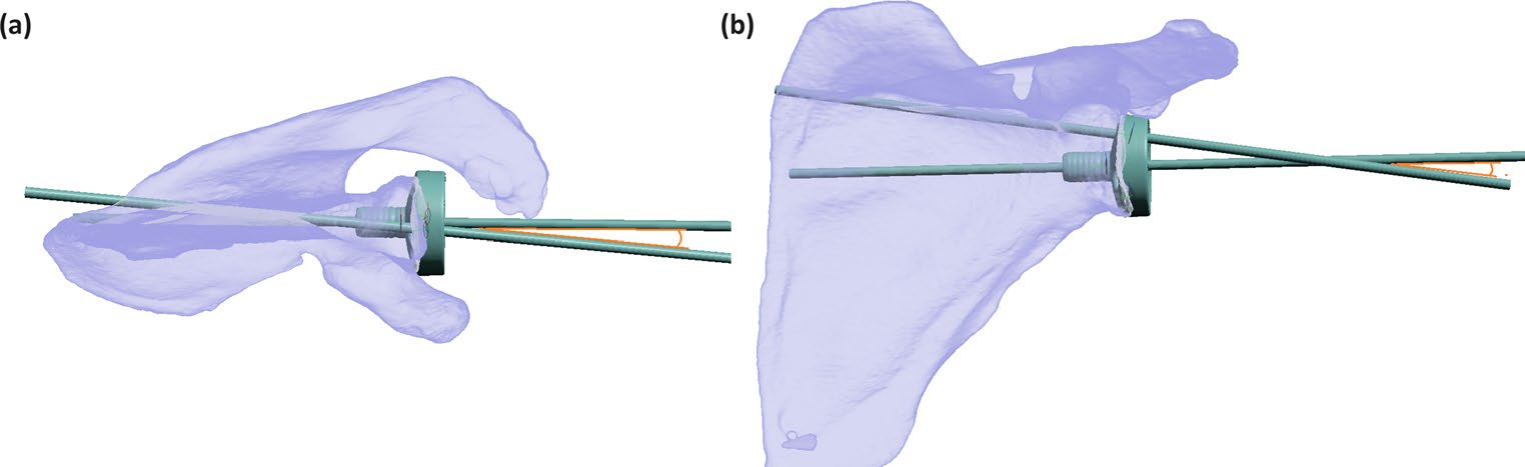
(**a**) Three-Dimensional view from above of the superior glenoid vault demonstrating the posterior orientation (6.5 degrees) in the optimal trajectory of the superior screw (blue), attributable to the relative posterior position of the scapular spine to the glenoid and slight glenoid retroversion relative to the scapular body. (**b**) AP view showing the optimal cranial angle of the superior screw (9.1 degrees)

**Fig. 5:**
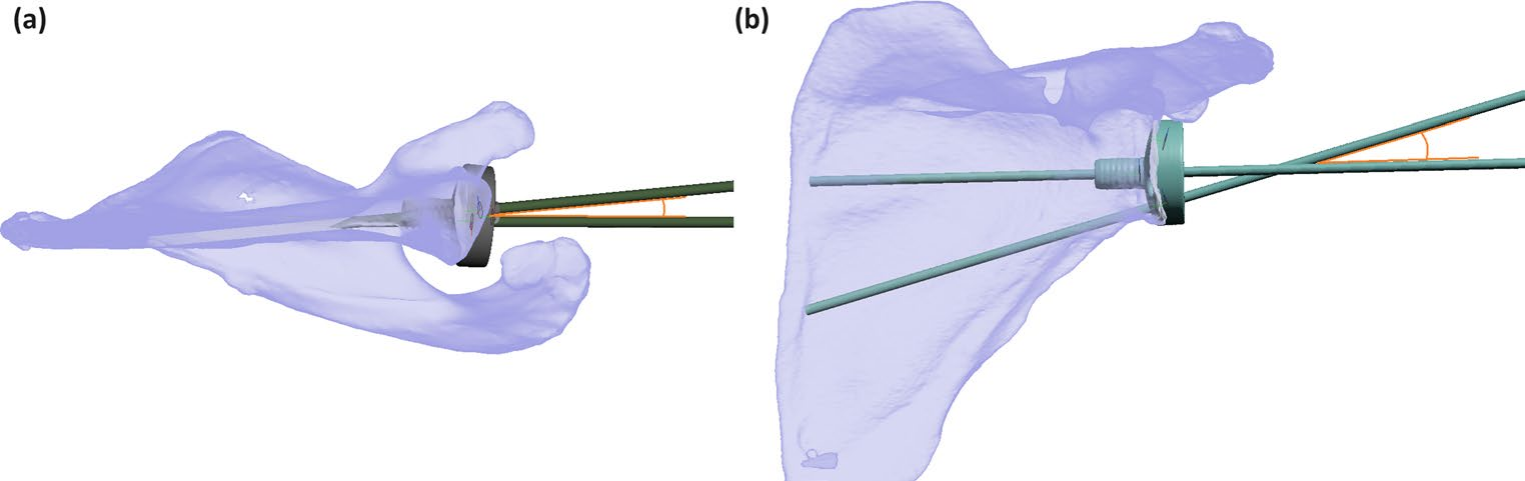
Three-Dimensional view from below of the inferior glenoid vault demonstrating the slight anterior orientation (0.7 degrees) of the optimal trajectory of the inferior screw (green) due to the retroversion of the glenoid relative to the scapular body. (**b**) AP view showing the optimal caudal angle of the superior screw (11.2 degrees)

With patient specific modelling, the average GRA value was − 1.6°±5.3 (range: -15.7-13), representing its rotation in the anterior direction. The optimal superior screw placement showed an average sCCA of 9.1°±4.0 (range: -2-19) cranial and an average sAPA of 6.5°±4.8 (range: -11.2-17.6) angulated posteriorly as shown in Fig. 4. For the inferior screw, optimal iCCA was 11.2°±4.0 (range: 3.5–24.7) caudal and iAPA of ---0.7°±5.6 (range: -11.8-16.0) as shown in Fig. 5. The optimal bi-cortical screw lengths obtained were on average 51.3 ± 21.4 mm (range: 22.7–118) and 48.5 ± 8.6 mm (range: 24.9–84.5) for the superior and inferior screws respectively. There was a positive Pearson correlation between glenoid anterior-posterior diameter with both superior (*R* = 0.249, *p* < 0.01) and inferior screw lengths (*R* = 0.215 *p* = 0.02). Screw lengths and age did not have statistically significant correlation. No statistically significant differences were observed between gender and glenoid roll angle nor superior and inferior screw angles except the superior screw cranial angle, with males being 11.7°±3.52 and females 8.11°±3.65 (*p* < 0.01). However, the differences amounted to the same rounded average scenario for validation testing.

Computational modeling using the CA for all angles in each case yielded an average screw length of 40.6 ± 18.4 mm (range: 16.9–98.3) and 38.2 ± 10.9 mm (range: 13.4–90.8) for superior and inferior screws respectively. Paired sample t-test comparing to PSI optimal screw lengths was statistically significant (p-value < 0.01) as shown in Table 2 and summary. With RA computational modelling, maximal screw lengths were on average 40.5 ± 18.1 mm (range: 15.1-103.3) and 39.4 ± 9.7 mm (range: 17.2–71.7) for superior and inferior screws respectively. Paired sample t-test comparing RA and PSI modelling showed statistical significance with a p-value < 0.01. No statistical significance was found between the CA and RA modeling superior and inferior screw lengths (*p* = 0.89, *p* = 0.05 respectively). All re-computation modeling of screw trajectories in the CA and RA groups did not perforate the suprascapular notch nor the spinoglenoid notch predisposing to suprascapular nerve injury.

**Table 2:** Mean maximal bi-cortical screw lengths attained from patient specific modelling compared to validation testing using calculated averages and rounded averages computer modelling

	Patient specific model	Calculated average model		Rounded average model		ANOVA test *p*-value
GRA (degrees)	PS	-1.6°		0°		**-**
Superior Screw Angles (degrees)	PS	sCCA: 9.1°	sAPA: 6.5°	sCCA: 10°	sAPA: 5°	**-**
Average Superior screw length (mm ± SD)	51.3 ± 21.4	40.6 ± 18.3		40.5 ± 18.1		**< 0.01**
Inferior Screw Angles (degrees)	PS	iCCA: 11.2°	iAPA: -0.7°	iCCA: 10°	iAPA: 0°	**-**
Average inferior screw length (mm ± SD)	48.5 ± 8.6	38.2 ± 10.9		39.4 ± 9.7		**< 0.01**

*PS: Patient specific, sCCA: Superior screw cranial angle, sAPA: Superior screw anterior-posterior angle (posterior as positive value), iCCA: Inferior screw caudal angle, iAPA: Inferior screw anterior-posterior angle (anterior as negative value)

## Discussion

The reverse shoulder arthroplasty developed by Grammont is a semi-constrained prosthesis with an increased deltoid fulcrum [16]. High stresses at the bone-prosthesis interface have led to catastrophic early failures. Obtaining strong early baseplate fixation is complicated by heterogenous glenoid geometry with different sizes and morphology. Data in Asian countries have shown an average glenoid width to often be less than the minimal metaglene diameter of various manufacturers (25–28 mm) especially in females [7, 14, 17]. A combined average of 28.2 *±* 4.5 mm of glenoid diameter was found in our study population, comparable to Western anatomy as demonstrated by Mathews et al. [18]. However, in contrast our population included more proportion of males and pathological glenoid morphologies with 23.4% and 13.7% of the cases having osteoarthritis and rotator cuff arthropathy respectively. Osteophyte and glenoid erosion may lead to an overestimation of glenoid diameter in our study population. Axial glenoid morphology have been described to have different configurations offsetting anterior or posterior screws resulting in little to or no bony purchase [19]. Roche et al. demonstrated that only two screws for baseplate fixation conferred more implant displacement [20]. However, longer screw lengths showed better fixation irrespective of screw quantity – highlighting the importance of attaining maximal screw length in glenoid baseplate fixation.

A previous cadaveric study investigated glenoid baseplate rotation and screw length comparing baseplate rotation at 12 o’clock and 20 degrees rotation anterior or posterior [21]. The screw lengths obtained in a perpendicular angle and variable angle with baseplate rotation in these 3 positions were assessed. Results showed rotation at neutral (12 o’clock) and rotation anteriorly provided the longest screw lengths. However, the optimal degree of rotation and screw angulation were not measured. In this study utilizing 3D model computation – we can accurately define the best glenoid baseplate angular rotation (GRA) and screw angulation in two planes (CCA and APA) for maximizing bi-cortical screw length.

However, validation testing of the calculated angles against patient specific measurements, demonstrated statistically shorter screw lengths attainable. This illustrates the substantial patient anatomical variation and heterogeneity. Therefore, the use of ancillary operative technology to help improve accuracy is of great importance in RTSA. This has been shown in navigation assisted RTSA with improved baseplate and screw configurations [13, 22–24]. As well as in 3D planning with patient specific instrumentation, which has been shown to be reproducible and accurate with minimal deviation from pre-operative planning [25–27]. Studies on the outcomes of robotic assistance in reverse shoulder arthroplasty is also on the horizon [28]. 

The limitation of this study includes the use of a computational model specifically with circular metaglene systems and variable angle screws located directly superior and inferior to the central peg. We did not evaluate the screw lengths of implant designs using screws perpendicular to baseplate. Further biomechanical studies are required to determine the minimal length of screws required to limit micromotion and allow stable initial fixation. A previous study compared 18 mm and 36 mm screw lengths which showed significant difference in micromotion for the anterior, superior and inferior screws in a poor density bone model [29]. Micromotion in the 18 mm screw trials exceeded 150 μm required for bone ingrowth. However, differing screw lengths for floor and ceiling effects were not investigated.

## Conclusion

In conclusion, the optimal average glenoid baseplate rotation was − 1.6°, close to perpendicular to the glenoid long axis. While optimal superior screw angulation was 9.1° cranial and 6.5° posterior. Inferior screw angulation of 11.2° caudal and 0.7° anterior, close to perpendicular to the glenoid plane. However, validation shows strong patient heterogeneity and anatomical variation. Nonetheless, as demonstrated from the pragmatic, rounded averages simulation – long screw lengths of > 38 mm for both superior and inferior screws were attainable with a good safety profile. Surgeons may consider the additional use of navigation-assisted, or 3D printed patient specific instrumentation to optimize baseplate and screw configuration in reverse shoulder arthroplasty.

## Electronic supplementary material

Below is the link to the electronic supplementary material.

Supplementary Material 1

## Data Availability

Not applicable.
