# Supplementary figures and images for: Computer modeling and validation testing for glenoid component rotation and optimal glenoid screw angles for reverse shoulder arthroplasty in an Asian population
Source: Int Orthop. 2024 Sep 30;48(12):3151–7. doi: 10.1007/s00264-024-06340-z (PMC11564314; doi:10.1007/s00264-024-06340-z)

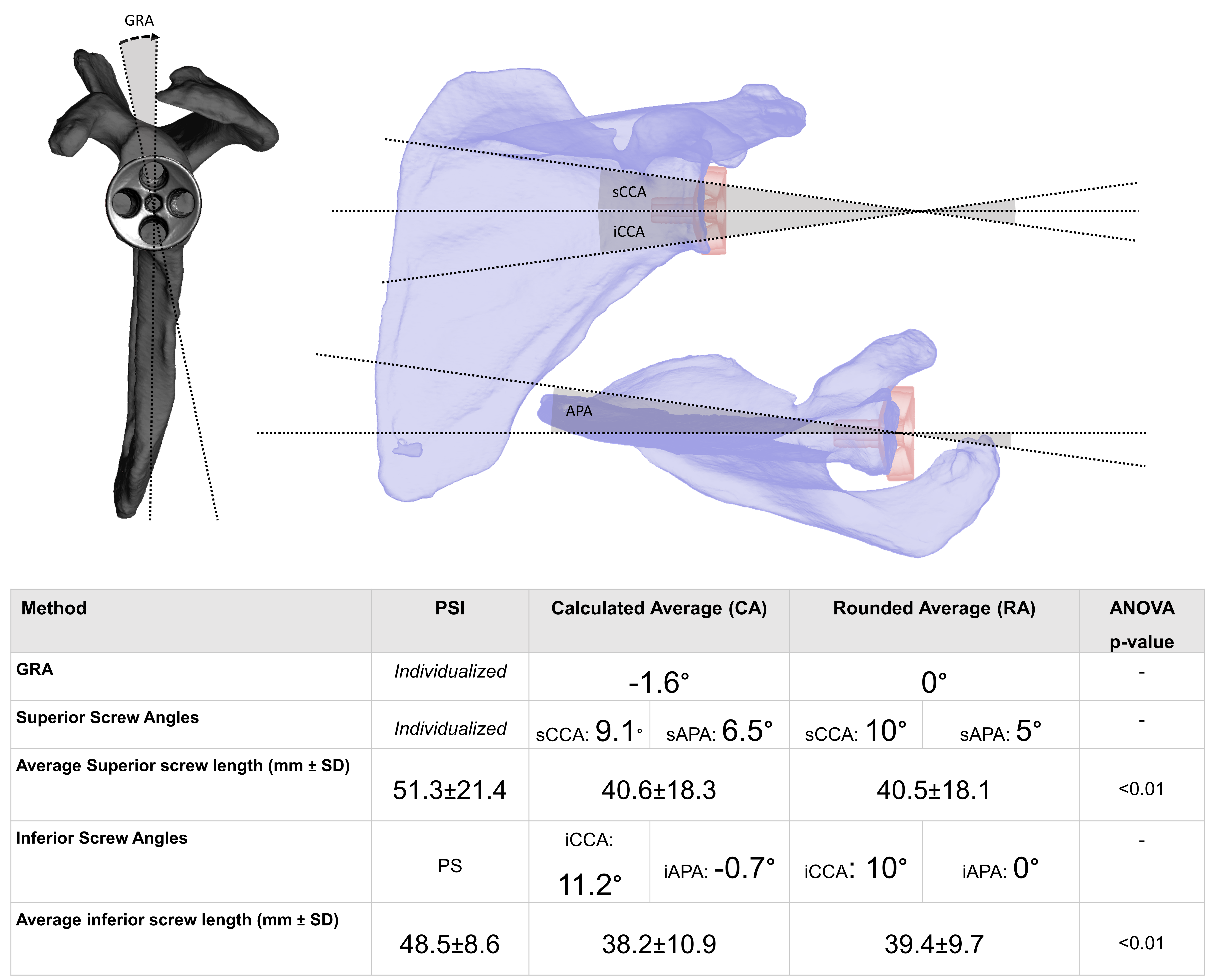

Supplement: Supplementary file 1 — Supplementary Material 1 [file 264_2024_6340_MOESM1_ESM.png]
